# Metabolites of endophytic fungi isolated from leaves of *Bauhinia variegata* exhibit antioxidant activity and agonist activity on peroxisome proliferator-activated receptors α, β/δ and γ

**DOI:** 10.3389/ffunb.2022.1049690

**Published:** 2022-12-08

**Authors:** Pedro Góes Mesquita, Laiza Magalhaes de Araujo, Francisco de Assis Rocha Neves, Maria de Fátima Borin

**Affiliations:** Laboratory of Molecular Pharmacology, Department of Pharmacy, Faculty of Sciences Health, University of Brasilia, Brasilia, Brazil

**Keywords:** peroxisome proliferator-activated receptors, antioxidant, diabetes, endophytic fungi, *Bauhinia variegata*

## Abstract

*Diabetes mellitus* is a metabolic disorder that affects millions of people worldwide and is linked to oxidative stress and inflammation. Thiazolidinediones (TZD) improve insulin sensitization and glucose homeostasis mediated by the activation of peroxisome proliferator-activated receptors γ (PPARγ) in patients with type 2 diabetes. However, their use is associated with severe adverse effects such as loss of bone mass, retention of body fluids, liver and heart problems, and increased risk of bladder cancer. Partial PPARγ agonists can promote the beneficial effects of thiazolidinediones with fewer adverse effects. Endophytic fungi colonize plant tissues and have a particularly active metabolism caused by the interaction with them, which leads to the production of natural products with significant biological effects that may be like that of the colonized plant. Here, we identify seven endophytic fungi isolated from *Bauhinia variegata* leaves that have antioxidant activities. Also, one of the extracts presented pan-agonist activity on PPAR, and another showed activity in PPARα and PPARβ/δ. A better understanding of this relationship could help to comprehend the mechanism of action of antioxidants in treating diabetes and its complications. Moreover, compounds with these capabilities to reduce oxidative stress and activate the receptor that promotes glucose homeostasis are promising candidates in treatment of diabetes.

## 1 Introduction

*Diabetes mellitus* is a metabolic disorder characterized by chronic hyperglycemia with disturbance in the metabolism of carbohydrates, lipids, and proteins. It results from a deficiency in insulin secretion, insulin action, or both, causing glucose to be unavailable in the muscles and adipose tissue and increasing gluconeogenesis in the liver (Alberti and Zimmet, 1998). Chronic hyperglycemia causes major vascular complications and is involved in developing heart disease, retinopathy, kidney disease, and circulatory problems. The development of diabetes can occur by the autoimmune destruction of beta cells, leading to a deficiency in insulin production, or by other factors that can result in tissue resistance to the action of insulin. In type 2 diabetes, insulin secretion and action deficiency are usually present in the same individual, which is the leading cause of hyperglycemia (Ada, 2014). When there is a clinical manifestation, individuals usually have insulin resistance, insulin deficiency, or both. The specific causes of type 2 diabetes are not yet defined; however, most patients are obese or have a high concentration of abdominal fat. Obesity causes a continuous elevation in plasma levels of free fatty acids, which induces hyperglycemia and impairs insulin sensitivity. The elevation of insulin secretion is a compensatory mechanism for maintaining glycemia. However, very high insulin levels do not allow compensation for the deficiency in glucose uptake and lead to insulin resistance and diabetes. Furthermore, obesity, recent weight gain, and the length of time one have been obese influence the increased risk of insulin resistance (Alberti and Zimmet, 1998; Felber and Golay, 2002; Ada, 2014).

In obesity, weight gain occurs when calorie intake exceeds energy expenditure when there is no energy balance, and the excess is stored in adipose tissue in the form of fat (lipids). The influence of adipose tissue on metabolic syndrome is central. First regarded as an energy storage tissue, it is now known that adipose tissue plays a fundamental role as an endocrine organ, capable of secreting molecules such as leptin and influencing insulin resistance, inflammation, eating behavior, and energy expenditure. It can communicate with other tissues in the body, such as muscle, liver, and the hunger center in the brain, and is in visceral and subcutaneous deposits. The accumulation of visceral adipose tissue (VAT) is linked, as well as type 2 diabetes, to other pathologies, such as heart disease, kidney, lung, and liver dysfunction, dyslipidemia, and hyperglycemia. In subcutaneous adipose tissue (SAT), it is associated with a low risk of these diseases (Majka et al., 2011).

Chronic systemic inflammation in adipose tissue is directly associated with the development of insulin resistance and type 2 diabetes, a feature of the chronic hyperglycemic state (Xu et al., 2003; Oguntibeju, 2019). Treatment usually is performed with diet, physical exercise, and antihyperglycemic agents using the following classes of drugs: *biguanides*, which suppress the production of glucose in the liver; *glitazones*, which improve insulin sensitivity by binding to PPARγ and increasing adiponectin signaling pathways; *sulfonylureas* and *glinides*, which stimulate insulin secretion; *GLP1 receptor agonists* (glucagon-like peptide 1), which mimic incretins, potentiating glucose-stimulated insulin secretion, decreasing glucagon secretion, promoting a feeling of satiety and decreasing gastric emptying; *gliptins* that potentiate the action of incretins and prevent the degradation of GLP1 by inhibiting DDP-4 (dipeptidyl peptidase 4); and *alpha-glucosidase inhibitors* that decrease glucose absorption in the intestine (Marín-Peñalver et al., 2016).

Oxidation and free radicals are part of the natural physiological process in multicellular organisms. Free radicals are molecules, ions, or atoms that have an unpaired electron, increasing their ability to react with other molecules. There is a very fragile balance between the production of radical compounds and their neutralization by antioxidant compounds. An imbalance known as oxidative stress occurs, which usually leads to physiological dysfunction (Halliwell, 2011). Oxidative stress is present in most processes involved in the development of diabetes. In the pancreas, the increase in reactive oxygen species (ROS) production by insulin-producing β-cells can occur through several pathways, including increasing the excessive concentration of glucose and free fatty acids (King and Loeken, 2004; Evans et al., 2005; Lupi et al., 2007; Zunino, 2009). The regulatory mechanisms involved in the pathological processes of diabetes and atherosclerosis in a correlation between oxidative stress and activation of peroxisomal proliferating receptors have been shown (Devchand et al., 2004; Hwang et al., 2004; Teissier et al., 2004; Jiang et al., 2009; Martin, 2010; Guellich et al., 2013). Polyphenols are the most abundant antioxidant substances in the diet and can be produced by plants and fungi (Manach et al., 2004). Flavonoids are polyphenols with diverse physiological and pharmacological activities, such as estrogenic, antitumor, antimicrobial, antiallergic, anti-inflammatory, and the well-known antioxidant and metal chelation (Cotelle, 2001; Reiterer et al., 2004; Das and Rosazza, 2006; Nazari et al., 2011). These substances have shown a beneficial effect concerning *diabetes mellitus*, acting in several ways, such as regulating glucose biochemical pathways, glucose tolerance, lipid profile, glycogen biosynthesis, glucose uptake, and insulin release (Volpato et al., 2008; Da Cunha et al., 2009). In addition, a meta-analysis published showed that dietary flavonoid intake was associated with a decrease in type 2 diabetes (Liu et al., 2014).

New drugs with better efficacy in diabetes treatment would benefit a large population. In this endeavor, endophytic fungi are promising. They are microorganisms belonging to several orders of fungi, which colonize the living and internal tissues of plants, intracellularly or extracellularly, in a relationship that varies from symbiosis to the borderline of pathogenicity (Saikkonen et al., 1998; Gallo et al., 2007). These organisms usually produce a wide variety of organic compounds due to the close metabolic interaction with their hosts, which results in a particularly active secondary metabolism (Gallo et al., 2007). In some cases, the host plant and the fungus can produce the same metabolites, as shown by the endophytic fungus *Taxomyces andreanae*, capable of producing the anticancer taxol (Paclitaxel^®^), also produced by its host, *Taxus brevifolia* (Stierle et al., 1993).

*Bauhinia* sp. plants are used in traditional and folk medicine and are reported to possess antidiabetic and anti-inflammatory activities (Zakaria et al., 2007; Prabhakar and Doble, 2008; Frankish et al., 2010). Here, we identified seven endophytic fungi isolated from *Bauhinia variegata* leaves and evaluated their ethyl acetate (EtOAc) extract activities over PPARα, PPARβ/δ, and PPARγ receptors and their antioxidant profile.

## 2 Material and methods

### 2.1 Endophytic fungi isolation and culture

*Bauhinia variegata* leaves were collected in Brasília, DF, in July. Leaves were cleaned and disinfected, and endophytic fungi were isolated according to the methodology described by Larran e coworkers (2007). Isolated fungi were grown at Sabouraud-dextrose agar medium in slants. After five days of incubation at 30°C, cultures were transferred to 500 mL flasks containing 50 mL of pre-fermentative medium described by Jackson et al. (1993), incubated at 28°C, on an orbital shaker incubator at 150 rpm for 48 h. After this period, cultures were transferred to 2000 mL flasks containing 500 mL of fermentative medium and cultured for 72 h, at 28°C, with an orbital stirring of 150 rpm.

### 2.2 Extract production

The mycelial mass was separated from the submerged culture by filtration under a vacuum. The fluids of the cultures were then submitted to a liquid-liquid extraction using ethyl acetate as solvent (3 times the volume) to extract the fungi metabolites. The extract was then concentrated by evaporation under a vacuum and dissolved in 1 mL of methanol. These extracts were used to evaluate antioxidant activity and polyphenols and flavonoid contents.

### 2.3 DNA extraction and amplification of the ITS1-5,8S-ITS2 rRNA sequences

Endophytic fungi isolated from *Bauhinia variegata* leaves were identified by their DNA ribosomal sequences after amplification by polymerase chain reaction (PCR) technique.

Fungi were cultured in Yeast Peptone Dextrose medium, as previously described by (White et al., 1990) at 30°C for 48 hours in a rotating shaker at 120 rpm. Two grams of the cultured fungi were submerged in liquid nitrogen, powdered, and transferred to microtubes. The samples were added with 500 µL Tris buffer, pH 8, containing 10 mmol.L^-1^ EDTA and 2% SDS, followed by vortexing. Genomic DNA was isolated by the phenol/chloroform method. After that, isolated DNA was solubilized in deionized water and quantified according to the method described by Sambrook et al. (1989). The portion 5.8S of the ribosomal RNA was amplified using the primers of the conserved regions ITS1-ITS2 and ITS3-ITS4 (White et al., 1990). Amplification was carried out using a final solution reagent volume of 50 µL, containing 1 µL of genomic DNA (500 ng), 2.5 µL DNTP (4 mmol.L^-1^), 0.5 µL Taq polymerase (0.5 U.µL^-1^), 5 µL of Tris/HCl buffer (100 mmol.L^-1^ pH 8.0), 2 µL 50 mmol.L^-1^ MgCl_2_, 1 µL of each universal primer, ITS1-ITS2 or ITS3-ITS4 (21 pmol.L^-1^), and 39 µL of deionized water. Thermocycler was programmed with initial DNA denaturing cycle at 94°C for 2 minutes, followed by cycling at 94°C for 1 min, 54°C for 1 min, 72°C for 1 min, and ending with a final step at 72°C for 10 min (Guimarães et al., 2008). PCR products were purified using a QIAquick^®^ PCR Purification Kit, and their sequences were analyzed by Macrogen Inc. in the Republic of Korea. Either genomic DNA or PCR amplification products were analyzed in agarose 1% electrophoresis to determine the chain size (Möller et al., 1992), using 1kb plus DNA ladder (Life Thecnologies^®^) as a marker.

### 2.4 Phylogenetic analysis

PCR amplicons sequences were edited at BioEdit and compared, *via* BLAST, to sequences deposited in the GenBank database of the NCBI (National Center for Biotechnology Information). The sequences obtained and selected from GenBank (NCBI) were submitted to multiple alignments using the Clustal-W tool in the MEGA 11.0.13 software. The similarity was analyzed by grouping the neighbor-joining method with 1,000 bootstrapping repetitions to construct the phylogenetic tree (Tamura et al., 2021).

### 2.5 Determination of total polyphenolic content

Total polyphenols were quantified by the method described by Kumazawa et al. (2002) with minor modifications. For that, 76.8 µg of each extract was added to 250 µL of 10% sodium carbonate and 250 µL of Folin-Ciocalteu 1N. The final reaction solution volume was completed to 2.5 mL with distilled water, and the solution was vortexed. Phenols react with the Folin-Ciocalteu reagent producing a bluish-grey complex. After 60 minutes, absorbance was measured in a spectrophotometer at 760 nm. Gallic acid was used as standard, and results were expressed as gallic acid equivalents by extract mass (EAG.mg^-1^).

### 2.6 Determination of total flavonoid content

Determination of total flavonoid content was carried out according to the method described by Chang et al. (2002), based on forming an acid-stable yellow complex between aluminum chloride and flavones/flavonoids. In brief, 125 µL of 5% aluminum chloride in a methanol solution was added to 20 µL of the sample. The final volume was adjusted to 2.5 mL with methanol and vortexed. After 30 minutes, the absorbance was measured in a spectrophotometer at 425 nm. Quercetin was used as standard, and results were expressed as quercetin equivalents by mass of extracts (EQ.mg^-1^).

### 2.7 *In vitro* antioxidant activity determination

#### 2.7.1 DPPH· assay

The antioxidant activity of the fungi extract was determined using DPPH· (2,2-diphenyl-1-picrylhydrazyl) assay, as described by Blois (1958). Negative control was obtained by replacing the sample with the same volume of methanol in the reaction solution. Antioxidant activity, determined by measurement of the percentage of reduction of DPPH·, considering the absorbance of the negative control as 100%, was estimated based on the formula below:


$$AA=\left[\frac{ABS_{control}-ABS_{sample}}{ABS_{control}}\right]\cdot 100$$

Where:

AA: antioxidant activity (%)

ABS_control_: absorbance of the negative control at 517 nm.

ABS_sample_: absorbance of the sample at 517 nm.

The percentage of antioxidant activity was evaluated by comparing the results obtained for different concentrations of extracts plotted in a graphic and calculating the IC_50_ (concentration of the extract that decreases by 50% the oxidation of DPPH·) (Dinis et al., 1994).

#### 2.7.2 Evaluation of the capability of the extracts in inhibiting lipid peroxidation

To evaluate the capability of the extract to inhibit lipid peroxidation, the method described by Lingnert et al. (1979) was used with some modifications. Briefly, linoleic acid was emulsified with the same volume of Tween® 20 in 0.1 M phosphate buffer, pH 7.0. For 2 mL of linoleic acid/buffer emulsion, 100 µL of samples were added, and the reaction solution was incubated for 8 hours at 37°C, hidden from the light. Then, 100 µL of this reaction solution was solubilized into 1 mL of methanol and added with 3 mL of 60% methanol solution, stirred. The absorbance was determined at 234 nm in a spectrophotometer (Shimadzu UV/VIS Model-1800).

#### 2.7.3 Superoxide anion radical scavenging activity

The superoxide anion radical ( ${\text{O}}_{2}^{-}\cdot$ ) scavenging activity by extracts was measured by determination of the inhibition of the NBT (Nitro Blue Tetrazolium) reduction. The xanthine/xanthine oxidase system was used as a superoxide anion radical source. The reaction was monitored by spectrophotometry at 560 nm for 10 minutes, modifying the method described by Robak and Gryglewski (1988). Results were expressed as sample concentration (mg.mL^-1^) able to inhibit 50% of the NBT oxidation rate acquired for the control reaction solution (IC_50_).

### 2.8 Cell culture

For the reporter gene assays, human mesangial cells donated by the Discipline of Nephrology at Escola Paulista de Medicina, Universidade Federal de São Paulo (UNIFESP) were used. Cells were cultured according to the method described by Chiellini and collaborators (Chiellini et al., 1998).

These cells were grown in Dulbecco’s Modified Eagle Medium (DMEM), containing 10% fetal bovine serum, 2 nmol.L^-1^ glutamine, 50 IU.mL^-1^ penicillin, and 55 IU.mL^-1^ streptomycin, and kept in 148 cm^2^ Corning^®^ plates in an incubator at 37° C with 5% CO_2_.

### 2.9 *In vitro* evaluation of the cytotoxicity of extracts on mesangial cells

Cell viability and cytotoxicity of the extracts were determined according to the 3-(4,5-dimethylthiazol-2- yl) -2,5-diphenyl-tetrazolium (MTT) method. The tetrazolium ring present in the yellowish-colored MTT bromide salt is cleaved by dehydrogenases present in the mitochondria of viable cells, forming blue-colored (Mosmann, 1983). Therefore, it is considered that the amount of viable cells is proportional to the amount of formazan crystals formed. In the assay, plates were seeded with 30,500 human mesangial cells per well in the DMEM culture medium, except for the well used as a reaction blank, which contained only the culture medium. The final reaction volume was 100 µL. After 24 hours of incubation at 37°C and 5% CO_2,_ 10 µL of MTT [5 mg.mL^-1^ in phosphate-buffered saline (PBS)] were added to all wells of the plates, and they were incubated for 4 hours, at 37°C and 5% CO_2_. For the dissolution of the formazan salt, the culture medium was removed from the wells, and 100 µL of MTT developer solution (0.04 mol.L^-1^ HCl in isopropanol) was added. The plates were left under agitation for 15 minutes at room temperature, and the absorbance was measured at 570 nm with a plate reader (Beckman Coulter DTX 800). The interpretation of the results was made by comparing the absorbance values obtained for cells treated with the extracts and those obtained in the controls.

### 2.10 PPAR receptor agonist activity – reporter gene assay

The reporter gene assay employing transient transfection was used in the research and identification of ligands for nuclear receptors (PPARα, PPARβ/δ, and PPARγ). This assay consisted of transfection by electroporation of expression and reporter plasmids inside the nucleus of human mesangial cells, followed by treatment of the cells with a known ligand (positive control) or with the extracts under study. In the presence of agonist substances, the reporter gene (luciferase) had its transcription increased and functioned as an indicator of the receptor’s transcriptional activity.

Transfection assay, cells were collected by centrifugation at 760.1 x *g* for 5 minutes and resuspended in PBS solution containing 0.1% dextrose and 0.1% calcium chloride to obtain a suspension containing 3x10^7^ cells.mL^-1.^ To each 500 µL of this solution of resuspended cells, 1.5 μg of the PPARα, PPARβ/δ, or PPARγ expression vector and 3 μg of the PPAR response element (PPRE) reporter plasmid vector were added. Cells were transferred to cuvettes and subjected to electroporation at 350 mV and 700 F using a Bio-Rad^®^ Gene Pulser II pulse generator. After electroporation, the cells were transferred to the culture medium, distributed in 12-well Corning^®^ plates (1 mL/well), and treated with vehicle, positive control, or different concentrations of the extracts based on the cytotoxicity assay.

After 24 hours of incubation at 37°C and 5% CO_2,_ cells were separated by centrifugation at 760.1 x *g* and lysed with 1X lysis buffer (Promega^®^). To determine luciferase activity, 20 μL of luciferin (Kit Promega Corp^®^) was added to 20 μL of cell lysate. The light emission generated by the enzymatic reaction between luciferin and luciferase was quantified in a luminometer GloMax^®^ 20/20, and the result was expressed in relative light units. All assays were performed in triplicate (Ribeiro et al., 2001). Data were expressed as the average transcription activation rate to the vehicle (DMSO:MeOH, 1:3) from at least four different experiments. Rosiglitazone 10^-5^ mol.L^-1^ was used as a positive control for assays using PPARγ, and bezafibrate at a concentration of 3x10^-4^ mol.L^-1^ was used as a positive control for assays performed with PPARα and PPARβ/δ. The results obtained were submitted to analysis of variance (one-way ANOVA) followed by the Newman-Keuls multiple comparison test.

### 2.11 Adipocyte differentiation assay

To assess whether the extracts produced by fungi BvFII and BvFVII were able to promote PPARγ-dependent 3T3-L1 preadipocyte differentiation and whether they interfered with differentiation promoted by PPARγ activation or with lipid accumulation within cells after differentiation, mouse embryonic fibroblasts (NIH 3T3-L1) were cultured in DMEM medium supplemented with 10% fetal bovine serum, 2 mmol.L^-1^ glutamine, 50 IU.mL^-1^ of penicillin and 50 IU.mL^-1^ of streptomycin and incubated at 36°C and 5% CO_2_. After the second day of confluent growth, cells were induced to differentiate by treatment with DMEM supplemented with 10% neonatal bovine serum, 1 μg.mL^-1^ insulin (Sigma, St Louis, USA), 0.5 mmol.L^-1^ 3-Isobutyl-1-methylxanthine (Sigma, St Louis, USA) and 1 mmol.L^-1^ dexamethasone (Sigma, St Louis, USA) for two days. After this period, the cells were treated with DMEM supplemented with 10% neonatal bovine serum and 1 μg.mL^-1^ of insulin and incubated at 37°C and 5% CO_2_ for an additional 2 days. After that, the cells were maintained in cultivation with DMEM supplemented with 10% fetal bovine serum. After two days of incubation, the cells were stained with Oil Red O as described by Janderová et al. (2003), to observe the differentiation. The vehicle, 10^-5^ M rosiglitazone, fungus BvFII extract, or fungus BvFVII extract were added to the culture medium during the differentiation induction period to observe their interference in the cell differentiation process.

#### 2.11.1 Extraction of total RNA from cells

After the treatment for the differentiation of adipocytes, the total RNA of the cells was extracted using the TRIzol reagent^®^, according to the protocol of the manufacturer Invitrogen™ (CA, USA).

#### 2.11.2 Quantitative evaluation of the expression of the gene encoding FABP4 by RT-qPCR

RNA samples extracted from adipocyte cultures were evaluated for the quantitative determination of the expression of the gene encoding the adipocyte protein fatty acid binding protein 4 (FABP4). FABP4 is regulated by PPARγ and is essential in adipocyte fatty acid uptake, transport, and metabolism. For the assay, reverse transcription and quantitative PCR amplification (RT-qPCR) was performed in a single step using the Power SYBR^®^ Green RNA to CT 1-Step kit (Applied Biosystems, Foster City, CA, USA), following the manufacturer’s instructions. The amplification product of each sample was determined by the SYBR^®^ Green system. Reactions were prepared in 96-well plates (MicroAmp Optical, Applied Biosystems) to a final volume of 10 μL, containing 0.08 μL of RT Enzyme Mix (125x), 5 μL of SYBR^®^ Green RT-PCR Power Mix (2x), 0.2 μL (100 nmol.L^-1^) of each primer (aP2 - Forward: CCATCTAGGGTTATGCTCTTCA and Reverse: ACACCGAGATTTCCTTCAAACTG; and 18S - Forward: CGGACAGGATTGAGAGATTG and Reverse: CAAATCGCTGCACCAACTAA) to obtain an initial concentration of 5 μmol,L^-1^, and 1 to 5 ng of RNA from the samples. The reaction conditions were the same for all RT-qPCR experiments: 30 minutes at 48°C to carry out the reverse transcription reaction; 10 minutes at 95°C for activation of the DNA polymerase enzyme; 40 cycles of 15 seconds at 95°C for denaturation; 1 minute at 60°C for annealing and extension on StepOnePlusTMReal-Time PCR Systems (Applied Biosystems) and the data obtained were analyzed with the software StepOne v2.1 (Bustin et al., 2009). The relative quantification of the expression of the gene encoding aP2 was performed using the equation 2^-ΔΔCt,^ where ΔΔCt represents the difference between ΔCt_sample_ and ΔCt_control,_ and ΔCt of the sample or control expresses the difference between Cq of the gene that encodes aP2 (FABP4) and the reference gene (18S) obtained for the samples. The statistical test applied was the analysis of variance (one-way ANOVA) followed by the Bonferroni test.

#### 2.11.3 Western blotting assay

Differentiated 3T3-L1 cells were harvested from the culture plates, resuspended in 0.01 M phosphate buffer, pH 7.4, containing 0.9% NaCl (PBS), and centrifuged at 760.1 xg for 5 min at 4°C. The pellets were homogenized in lysis buffer [50 mmol.L^-1^ Tris buffer pH 7.5 with 150 M NaCl, 1% Triton X-100 and a diluted protease inhibitor cocktail (Sigma Aldrich, St. Louis, MO, USA). 1:200] and slowly stirred for one hour at 4°C. The total protein concentration was determined by the Bradford method (Bio-Rad Protein Assay^®^). To evaluate aP2 (FABP4) protein expression, 12.5 µg aliquots of protein were separated by SDS-PAGE, transferred to a PVDF membrane in a semi-dry vat (Bio-Rad, CA, USA), incubated with antibodies, and then immunodetected by chemiluminescence (ECL prime kit, GE Healthcare, Buckinghamshire, UK). The membrane was incubated overnight at 4°C with the anti-aP2 polyclonal antibody (195657, Abcam, Cambridge, USA) and the polyclonal antibody anti-GAPDH (9483, Abcam, Cambridge, USA) diluted in water containing 7% powdered milk. Immunostains were visualized using a secondary antibody conjugated to anti-goat peroxidase IgG-horseradish peroxidase conjugate (HRP) (sc-2354, Santa Cruz, CA, USA), diluted 1:7000 in water containing 7% powdered milk. The default marker used was the BenchMark™ pre-stained protein Ladder (Life Technologies, Grand Island, USA) according to the methodology described by Burnette (1981). The quantification of bands was performed with the ImageJ program version 1.46r (National Institute of Health, Bethesda, MD, USA) and expressed as arbitrary units (UA/GAPDH).

### 2.12 PPARγ nuclear receptor protection assay against protease digestion

The ligand-binding domain (LBD) of human PPARγ was expressed in bacteria (*Escherichia coli*, strain BL21) as a polyhistidine-tagged recombinant protein (His-LBD-PPARγ) and purified by cobalt affinity chromatography. The recombinant protein was incubated with rosiglitazone, with the extract produced by fungus BvFII, with the extract produced by fungus BvFVII, or with vehicle dimethyl sulfoxide (DMSO) in PBS buffer (0.01 mol.L^-1^, pH 7.4) for 30 minutes at room temperature. Then, it was incubated with distilled water or trypsin solution (100, 250, or 350 μg.mL^-1^) for 60 minutes, also at room temperature. The digestion reaction was stopped by adding denaturing sample buffer (100 mmol.L^-1^ Tris buffer, pH 6.8, containing 2% β-mercaptoethanol, 4% SDS, 4 mmol.L^-1^ EDTA, 20% glycerol, and bromophenol blue) and heating at 95°C for 10 minutes. The LBD proteolytic pattern of the protein was then analyzed by electrophoresis on a 12.5% polyacrylamide gel under denaturing conditions (Laemmli, 1970), followed by staining of the gel with the protein-dye Coomassie blue (Allan et al., 1992).

### 2.13 Evaluation of the inhibition of trypsin proteolytic activity by the extracts of fungi BvFII and BvFVII

To evaluate the ability to inhibit the proteolytic activity of trypsin on gelatin by extracts of fungi BvFII and BvFVII, a polyacrylamide gel (12.5%) containing 0.25% gelatin was prepared. Then, 100 µL of reaction medium with 10 µL of trypsin (200 µg.mL^-1^) in 80 µL of PBS buffer (0.01 mol.L^-1^, pH 7.4), and 10 µL of vehicle (DMSO), fungus BvFII extract (400 µg.mL^-1^), or fungus BvFVII extract (400 µg.mL^-1^) were incubated for 1 h at room temperature. After the incubation period, 20 µL of each sample were soaked in 6 mm diameter filter paper discs deposited on the gel and incubated for 60 minutes at room temperature. Proteolytic patterns were determined by the difference in intensity of the gel staining by Coomassie blue. After the decolorization of the gel, the intensity of the bands was measured using the Image J program, version 1.46r (National Institutes of Health, Bethesda, MD, USA) and expressed as a rate of inhibition regarding trypsin activity (Chiang et al., 2012).

### 2.14 Statistical analysis

All the experiments were performed in at least three different sets, and data were expressed as mean ± standard deviation (SD) of at least three measurements. IC_50_ values were calculated using Graphpad Prism^®^ version 9.0.0. Data obtained in the experiments were analyzed through the one-way analysis of variance (ANOVA) test followed by Bonferroni’s multiple comparison tests. All statistical tests were performed using the software Graphpad Prism^®^.

## 3 Results

### 3.1 Submerged culture of the fungi and extract obtained

The mass of the extracts produced was determined, and the yield was calculated to the volume of fluid culture extracted and the mycelial mass produced. Fungus BvFI produced the highest amount of mycelia mass (wet weight 62.19 g) and the BvFII the lowest amount of mycelial mass (wet weight 6.32 g) after submerged liquid culture. However, the higher yield of metabolites extracts were acquired from the cultures of fungi BvFII and BVFIX that produced 35.2 mg and 89.5 mg of extract, respectively.

### 3.2 Molecular identification of fungi

#### 3.2.1 Conserved regions of the ribosomal RNA gene (ITS - Internal transcribed spacer) from the fungal genomic DNA were amplified

Conserved regions of the ribosomal RNA gene (ITS - Internal transcribed spacer) from the fungal genomic DNA were amplified for molecular identification. The polymerase chain reaction amplification products were sequenced and compared with sequence data from species described in GenBank (National Institute of Health).

The alignment performed in GenBank using the advanced Blast tool showed identity above 98% in all analyzes performed. The *e-value* (*expected value*) is the probability of an alignment occurring by chance and is calculated based on the quality of the alignment performed. The lower the *e-value*, the higher the quality of the alignment performed.

**Figure 1:**
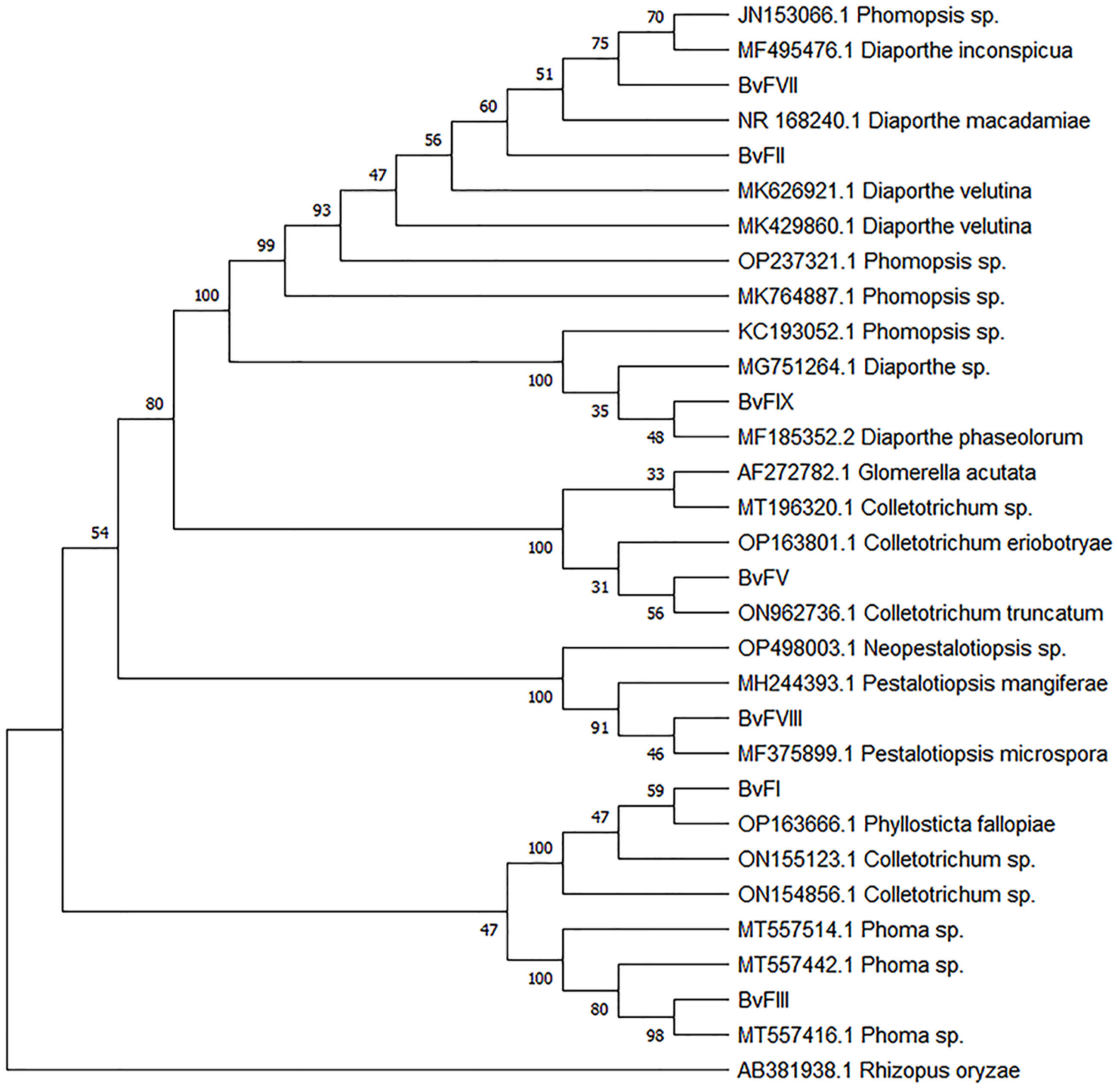
Phylogenetic tree constructed for PCR amplicons of the ITS1-5.8S-ITS2 rDNA of the endophytic fungi by genetic similarity comparison with data acquired with data from GenBank (NCBI). Phylogenetic tree constructed on the basis of the similarity of the PCR amplicons of the internal transcribed spacer 1 and 2 rDNA regions and sequences found in GenBank (NCBI), analyzed by the grouping neighbor-joining method, with 1,000 bootstrapping repetitions. The number of ramifications presents the bootstrap value (Felsenstein, 1985). The analysis involved 31 FASTA sequences. *Rhizopus oryzae* was used to root the phylogenetic tree. All positions containing gaps and missing data were eliminated. Evolutionary analyses were conducted in MEGA11 (Tamura et al., 2021).

As shown in the results, fungi BvFII, BvFVII and BvFIX belong to the same genus, *Phomopsis* sp., but the strains were kept separately because of their differences. Fungus BvFI showed a maximum identity of 94.20% with *Colletotrichum* sp. in the alignment performed. The fungus BvFV, with 100% identity, was characterized as *Colletotrichum* sp. Fungus BvFIII obtained greater identity with the fungi of the *Phoma sp* family, with a maximum identity of 99.64%. Fungus BvFVIII has a maximum identity of 100% with *Pestalotiopsis mangiferae*. The similarity was analyzed by grouping the neighbor-joining method with 1,000 bootstrapping repetitions to construct the phylogenetic tree (**Figure 1**).

### 3.3 Polyphenols and flavonoid contents

**Figure 2:**
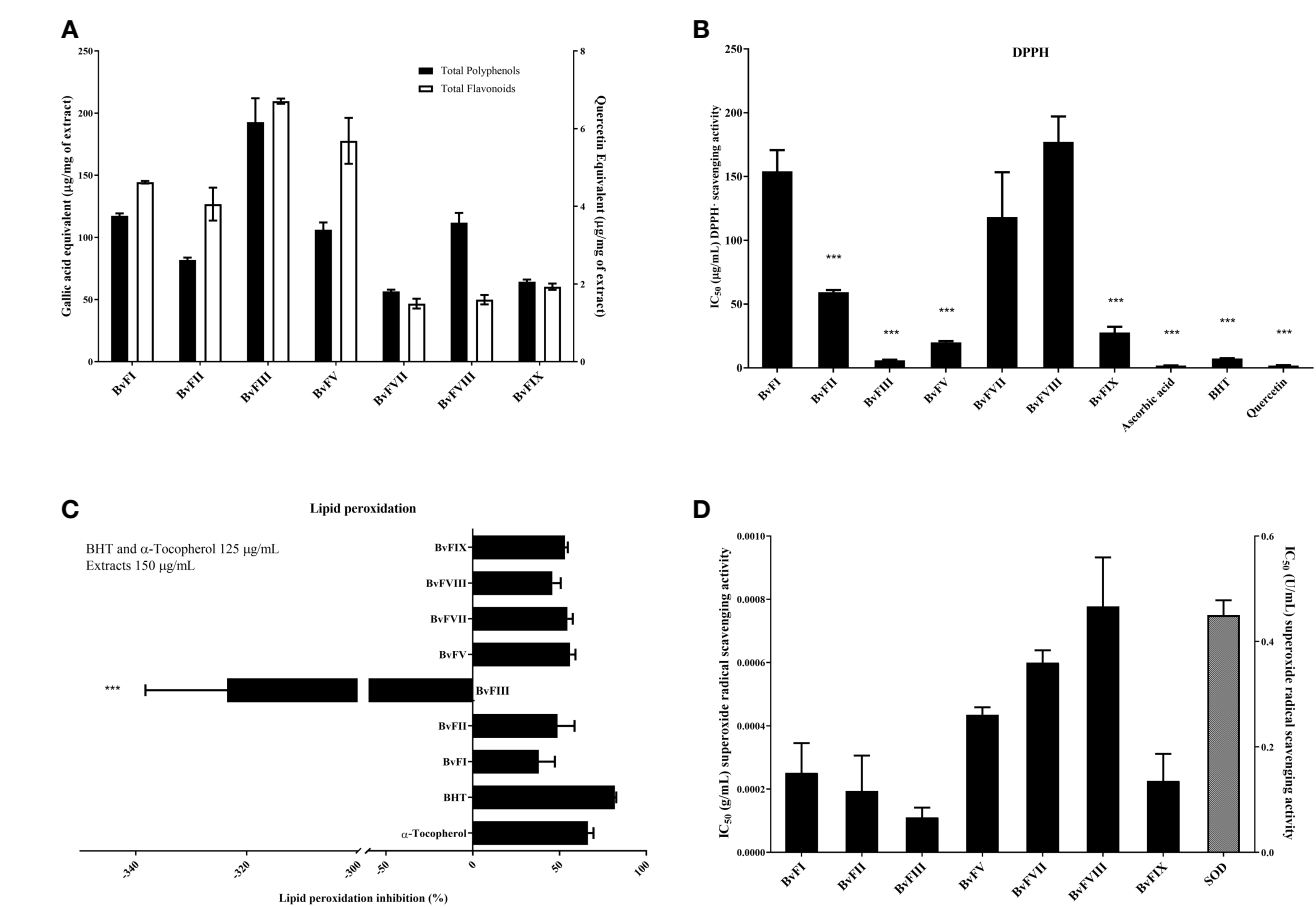
Polyphenols and flavonoids content and antioxidant effects. **(A)** Polyphenols and flavonoids contents in the extracts produced by the endophytic fungi isolated from *Bauhinia variegata* leaves. **(B)** Hydrogen-donating ability of the fungi extracts (BvFI, BvFII, BvFIII, BvFV, BvFVII, BvFVIII, and BvFIX) was evaluated by the DPPH· method. The concentration range of the extracts used in the reaction medium was 0.036 to 150.6 µg.mL^-1^. Standard compounds, ascorbic acid, BHT, and quercetin concentrations in the reaction medium also were used in the same concentrations. **(C)** Evaluation of the ability to inhibit lipid peroxidation by extracts of fungi. The emulsion of linoleic acid with polysorbate 20 in the presence and absence of extracts (150 µg.mL^-1^) and standards with the antioxidant activity known [tocopherol (125 µg.mL^-1^) and BHT (125 µg.mL^-1^)]. **(D)** Endophytic fungi extract ability to scavenge superoxide anion radicals. Extracts’ ability in scavenge superoxide anion radicals was evaluated using the xanthine/xanthine oxidase system as the source of the superoxide. ${\text{O}}_{2}^{-}\cdot$ produced reduces NBT to formazan detected by spectrophotometry at 560 nm. Antioxidants scavenge superoxide anion radicals, decreasing the reduction of NBT and, consequently, formazan production. Results were expressed as the percentage of inhibition of the lipid peroxidation process, as mean ± standard deviation of three different assays. The statistical test applied was the analysis of variance (one-way ANOVA) followed by the Newman-Keuls multiple comparison test, ***, significantly different, p<0.0001 vs. all compounds.

Obtained extracts were analyzed to determine their polyphenols and flavonoid contents using, respectively, Folin-Ciocalteu and aluminum chloride methods, as previously described. Calibration curves were constructed using gallic acid and quercetin as standards. Results are shown in **Figure 2A**.

### 3.4 *In vitro* antioxidant activity determination

#### 3.4.1 DPPH· assay

The antioxidant activity of the extracts was evaluated by DPPH· method and compared with compounds with recognized antioxidant activity, ascorbic acid, Butylhydroxytoluene (BHT), and quercetin. Results are reported in **Figure 2B**. Extracts of the fungi BvFIII, BvFV, BvFIX, and BvFII showed the higher DPPH· scavenging activity, the lower IC_50_ values in crescent order. The IC_50_ of the metabolites of fungus BvFIII was between those found to the standard antioxidants used as control, exhibiting a high antioxidant acitivity.

#### 3.4.2 Lipid peroxidation inhibition assay

Linoleic acid is a polyunsaturated fatty acid necessary for producing arachidonic acid. It is present in the cell membrane’s phospholipid and is involved in signaling as a lipid second messenger. Both lipids are PPAR ligands (Forman et al., 1997). To evaluate the influence of endophytic fungi extracts on lipid peroxidation, an emulsion of linoleic acid (5 mmol.L^-1^) and polysorbate 20 (Tween^®^20) in saline phosphate buffer (0.1 mol.L^-1^ and pH 7.0) was incubated for 8 hours at 37°C in the presence or absence of extracts and positive controls tocopherol and BHT. In this way, linoleic acid undergoes auto-oxidation, and its product can be measured by spectrophotometry at the absorbance of 234 nm. The results obtained in this test are shown in **Figure 2C**. Excepting by extract of fungus BvFIII, all the extracts were able to inhibit lipid peroxidation. The weakest effect was observed for the extract of fungus BvFI, with 38% inhibition of lipid peroxidation. The highest effect was observed for the extract of fungus BvFV, which inhibits 56.1% of the oxidation process. BHT and α-tocopherol used as positive controls presented 89.9% and 66.6% of lipid peroxidation inhibition, respectively. Extracts of fungi BvFII, BvFVII, BvFVIII, and BvFXI presented percentages of lipid peroxidation of 48.8%, 54.6%, 45.9%, and 53.2%, respectively. Results also suggested that the fungus BvFIII extract improved lipid peroxidation by more than 2 and a half times (-262.4%).

#### 3.4.3 Sequestration of the superoxide radical anion ( ${\text{O}}_{2}^{-}.$ )

Extracts’ ability in scavenge superoxide anion radical was evaluated using the xanthine/xanthine oxidase (Robak and Gryglewski, 1988) system as the source of the superoxide radical anion (O2-∙) produced reduced NBT to formazan detected by spectrophotometry at 560 nm. Antioxidants scavenge superoxide anion radicals, decreasing the reduction of NBT and, consequently, formazan production. Endophytic fungi extracts scavenged superoxide anion radicals (**Figure 2D**). Extract of fungus BvFVII (IC_50_ 0.80 mg.mL^-1^), fungus BvFVIII (IC_50_ 0.147 mg.mL^-1^) and BvFV (IC_50_ 0.083mg.mL^-1^) showed lowest ability in scavenge superoxide. Extracts of fungus BvFI (IC_50_ 0.208 mg.mL^-1^) and BvFIX (IC_50_ 0.199 mg.mL^-1^) presented similar activity and extracts of fungus BvFII and BvFIII, with IC_50_ 0.147 mg.mL^-1^ and 0.083 mg.mL^-1^, respectively, were the extracts showing highest ability in scavenge superoxide anion radical.

### 3.5 Adipocyte differentiation assay

**Figure 3:**
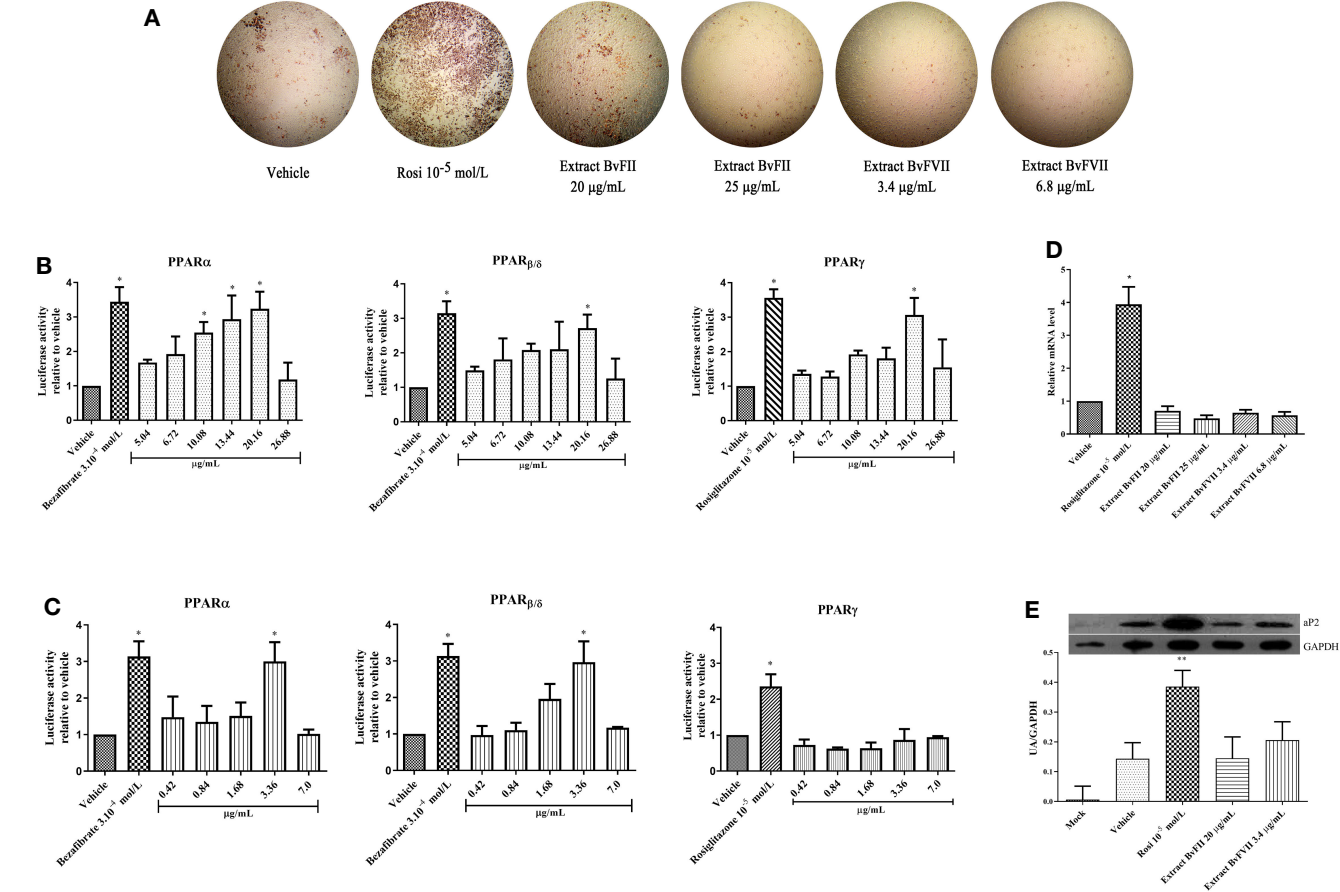
Extracts BvFII and BvFVII do not promote adipogenesis in 3T3-L1. Cells were induced to differentiate with insulin and exposed to vehicle (DMSO), Rosi 10^-5^ mol.L^-1^, Extract BvFII 20 or 25 µg.mL^-1^ and Extract BvFVII 3.4 or 7.8 µg.mL^-1^. **(A)** After 14 days, the cells were fixed, stained with oil red O, and photo documented. **(B, C)** Human mesangial cells were co-transfected with the expression vectors for PPARα or PPARγ, and the plasmid containing the luciferase reporter gene and treated with vehicle (DMSO:MeOH, 1:3), bezafibrate 3.10^-4^ mol.L^-1^for PPARα, β and rosiglitazone 10^-5^ M for PPARγ (positive controls) or increasing concentrations of extracts of fungi BvFII **(B)** and BvFVII **(C)**. **(D)** Real-time quantitative PCR to evaluate the expression of Fabp4 (aP2) after 8 days of differentiation. **(E)** Western blot to evaluate the expression of FABP4 (aP2) protein after 14 days of differentiation. Data are presented as the mean (SD) of three independent experiments conducted in triplicate and expressed as activation relative to transcript levels in vehicle samples (DMSO). *p ≤ 0.05; **p ≤ 0.001.

As can be seen in **Figure 3A**, mouse embryonic fibroblasts (NIH 3T3L1) showed different patterns of oil red O staining after differentiation. Cells treated with rosiglitazone (10^-5^ M) showed the most accumulation of lipids. The fungus BvFII extract, at a concentration of 20 µg.mL^-1^, also stimulated the differentiation of these cells to a degree slightly higher than that presented by the vehicle and lower than that induced by rosiglitazone. At the concentration of 25 µg.mL^-1^ of extract, there was no significant change in the staining of cells to the control. The fungus BvFVII extract did not promote cell differentiation compared to the vehicle.

### 3.6 Evaluation of agonist activity of fungi extracts in PPAR α, β and γ on reporter gene assay

The cultured extracts were submitted to the evaluation of their agonist capacity of the PPARα, PPARβ, and PPARγ using the gene reporter assay and treated using the concentration obtained on MTT assay. The results of the coactivation obtained are shown in **Figures 3B, C**. The extract of fungus BvFII was able to activate all PPAR isoforms, and this activation was similar to positive controls at a concentration of 20.16 µg.mL^-1^ (**Figure 3B**). The extract of fungus BvFVII also showed agonist activity, but only on PPARα and PPARβ receptors (**Figure 3C**). The other extracts didn’t show any activation (data not shown).

### 3.7 Evaluation of the relative gene expression of adipocyte protein 2 (aP2 or FABP4) by quantitative PCR

3T3-L1 cells were differentiated for 8 days with a complete medium and treated with vehicle (DMSO), rosiglitazone (10^-5^ mol.L^-1^), fungus BvFII extract (20 or 25 µg.mL**^-1^**), or fungus BvFVII extract (3.4 or 7.2 µg.mL**^-1^**) during differentiation. The difference in ap2 protein gene expression (FABP4) was not significant between the vehicle (DMSO) and the fungal extracts. There was only a significant difference between protein expression in cells treated with rosiglitazone and the other treatments (**Figure 3D**).

#### 3.7.1 Western blotting

To evaluate the influence of extracts produced by fungi BvFII and BVFVII on the expression of aP2 in differentiated 3T3-L1 cells, the total protein of the cells was extracted, quantified, and analyzed by *Western Blotting assay* (**Figure 3E**). The concentrations of 20 µg.mL**^-1^** for the extract of the fungus BvFII and 3.4 µg.mL**^-1^** for the extract of the fungus BvFVII were chosen to evaluate the expression of the aP2 protein because they showed the highest activation over PPARγ in the reporter gene assay (**Figures 3B, C**).

### 3.8 Protease digestion protection assay

**Figure 4:**
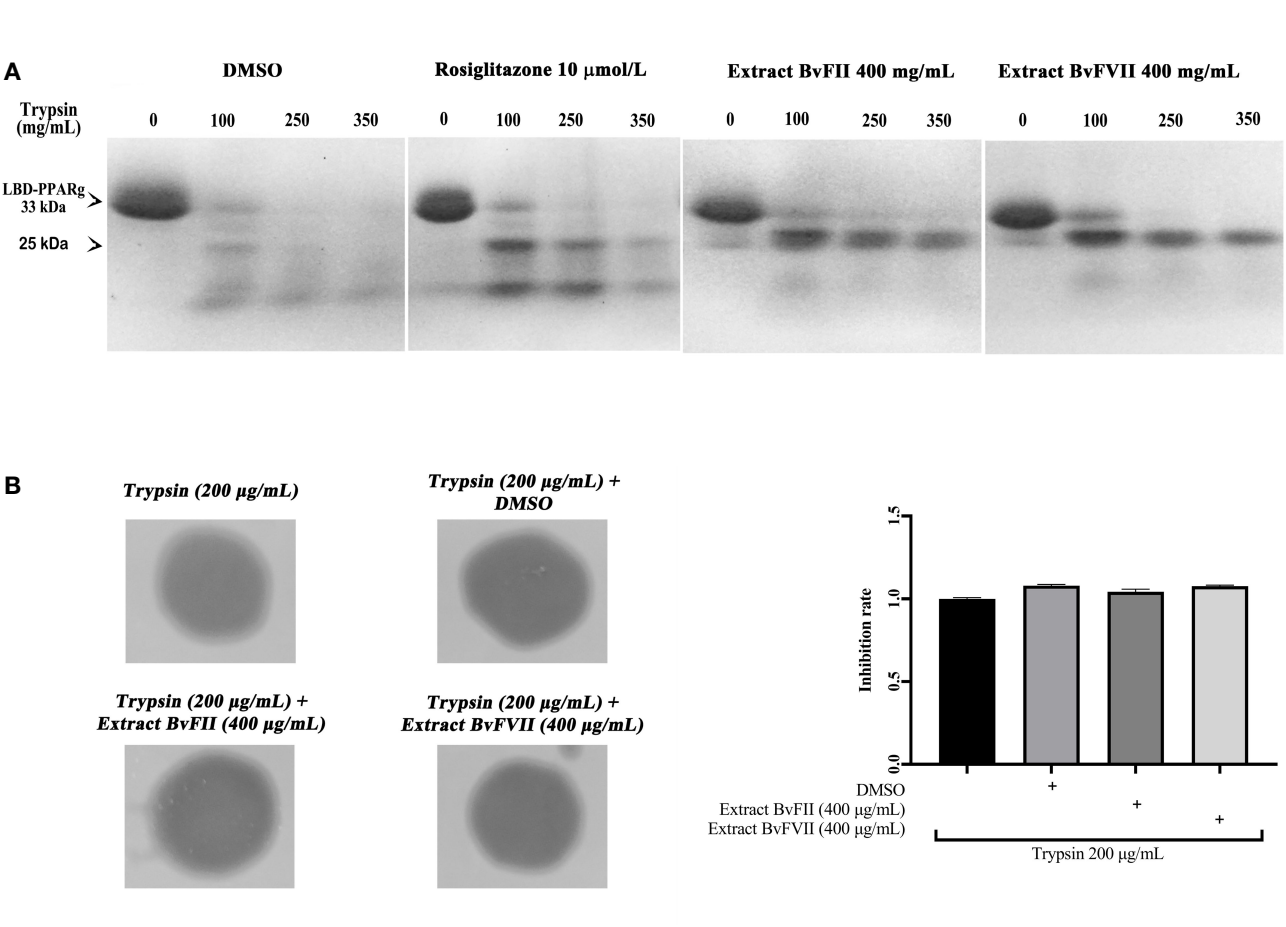
LBD proteolysis pattern of PPARγ by trypsin after treatment with different ligands. **(A)** Trypsin activity on PPARγ LBD was evaluated by using the recombinant protein his-LBD-hPPARγ, which was incubated with vehicle (DMSO), 10 µmol.L^-1^ rosiglitazone, fungus BvFII extract, or fungus BvFVII extract and with trypsin (0, 100, 250 and 350 μg.mL^-1^) for 60 minutes. LBD proteolytic patterns of PPARγ were analyzed by polyacrylamide gel electrophoresis under denaturing conditions, followed by staining the gel with Coomassie blue. **(B)** The proteolytic activity of trypsin on gelatin was evaluated after incubation of the enzyme with vehicle (DMSO) or with extracts of fungus BvFII (400 µg.mL^-1^) or fungus BvFVII (400 µg.mL^-1^). Polyacrylamide gel with 0.25% gelatin was left in contact with filter paper discs soaked in 20 µL of trypsin solution (200 µg.mL^-1)^ for 1 h. Proteolytic patterns were analyzed by staining the gel with Coomassie blue. Dark areas show degraded gelatin. The quantification of the inhibition rate of vehicle and extracts BvFII and BvFVII is shown on the right. The data presented represent the mean ± standard deviation of the mean. The statistical test applied was the analysis of variance (one-way ANOVA) followed by the Newman-Keuls multiple comparison test, with no statistically significant difference.

The binding of agonists to the LBD of nuclear receptors determines conformational changes critical for transcriptional activation by these receptors (Allan et al., 1992). These alterations are reflected in a modification of the accessibility of proteolytic sites on the surface of the receptor and, therefore, in an increase in the resistance of the receptor’s LBD to digestion by proteases. Thus, the protease digestion protection assay is one of the instruments used to study PPARγ conformational changes in the presence of ligands. From the results obtained, shown in **Figure 4A**, it is possible to observe that there was greater protection against digestion exerted by trypsin both for the samples treated with the extract of fungus BvFII and for those treated with the extract of the fungus BvFVII when compared to the treatment with rosiglitazone, even for the trypsin dose of 350 µg.mL^-1^.

### 3.9 Evaluation of the inhibition of trypsin proteolytic activity by extracts of fungi BvFII and BvFVII

Samples of the extract of fungus BvFII or fungus BvFVII were incubated with trypsin in PBS buffer to assess whether the extracts interfered with the proteolytic activity of the enzyme. Trypsin proteolytic activity was evaluated in a polyacrylamide gel containing gelatin (**Figure 4B**). As a negative control, trypsin was incubated with DMSO alone. The results suggest that extracts from fungus BvFII (400 µg.mL^-1^) or fungus BvFVII (400 µg.mL^-1^) do not interfere with the proteolytic activity of trypsin on gelatin.

## 4 Discussion

The potential of drugs from the microbiota of the Cerrado biome is little explored, and numerous studies have shown that fungi, especially endophytic fungi, emerge as a new perspective source of natural products with biological activity (Strobel et al., 2004; Zhang et al., 2006; Arnold, 2007; Rodriguez et al., 2009; Zhou et al., 2010; Blackwell, 2011; Mousa and Raizada, 2013).

*Bauhinia variegata* is a plant well adapted to the Cerrado and produces phenolic compounds, such as tannins and flavonoids (Duarte et al., 2007), and has anti-inflammatory (Mohamed et al., 2009), hepatoprotective activity (Bodakhe and Ram, 2007), antihyperglycemic (Azevedo et al., 2006; Kumar et al., 2012; Kulkarni and Garud, 2015) in addition to activating PPARγ (Silva, 2014).

After the publication of the work describing the ability of an endophytic fungus to produce the same active metabolite as its host plant (Stierle et al., 1993), the prospect was opened that natural products found in medicinal plants could be produced by endophytic fungi that colonize them (Newman and Cragg, 2015).

In this sense, by producing substances with hypoglycemic activity (Azevedo et al., 2006; Frankish et al., 2010; Kumar et al., 2012; Kulkarni and Garud, 2015) and substances that activate PPARγ (Costa, 2005; Silva, 2014), *Bauhinia variegata* was chosen for the isolation of endophytic fungi that colonize it, to evaluate the ability of these fungi to produce metabolites that could activate PPAR (α, β/δ and, γ) and that had antioxidant activity, to search for new bioactive compounds that could be used in the treatment of diabetes.

Three of the seven isolated endophytic fungi, BvFII, BvFVII, and BvFIX, were identified as fungi from the genus *Phomopsis* (**Table 1**). This genus corresponds to the asexual (anamorph) form of the ascomycete *Diaporthe*, which encompasses more than 900 species of fungi, and is characterized as a major producer of secondary metabolites, including antidiabetic, anti-inflammatory, and antioxidant compounds (Xu et al., 2021). The fungi BvFI and BvFV showed higher similarity with *Colletotrichum* sp. on the blast analysis. Tianpanich et al. (2011) isolated six substances with antioxidant activity from a *Colletotrichum* sp. that were characterized as five isocoumarin derivatives and a phthalide, of which four showed cytotoxic activity. In another work, Li et al. (2022) demonstrated that diterpene lactones obtained from *Colletotrichum* sp. showed strong antibacterial and also antioxidant activity. The fungus BvFI was related to the *Guignardia* (synonym: *Phyllosticta)* (**Figure 1**), an endophytic/phytopathogenic fungus. Some species of the genus *Guignardia*, such as *Guignardia citricarpa* and *Guignardia psidii* cause diseases such as citrus black spot and guava fruit rot. Other species like *Guignardia mangiferae*, *P. capitalensis*, and *P. citribraziliensis* are considered endophytic because they were extracted from plants without signs of infection. *Guignardia mangiferae* is an important producer of bioactive substances, such as terpenes (Yuan et al., 2010) and acetylcholinesterase inhibitors. The fungus BvFIII was characterized as *Phoma* sp. This genus is known as an important plant pathogen, and it is also found in the marine environment; it produces hundreds of secondary metabolites already described (Rai et al., 2018), among them substances with antifungal and cytotoxic action against tumor cell lines have already been described (Li et al., 2005; Wang et al., 2012). These previous works corroborate the results obtained in this work, which show the antioxidant activity of the metabolites of these fungi isolated from *B. variegata* (**Figure 2**).

**Table 1:** Strains of endophytic fungi isolated from *Bauhinia variegata*.

Fungus	GenBank access number	Consultation coverage	Identity maximum	*e-value*	Species (GenBank access number)
BvFI	OP604487	100%	94.20%	0.0	*Colletotrichum* sp. (ON154856.1)
BvFII	OP604488	99%	99.64%	8 x 10^-139^	*Phomopsis* sp. (OP237321.1)
BvFIII	OP604489	99%	100%	1 x 10^-126^	*Phoma* sp. (MT557416.1)
BvFV	OP604490	99%	100%	3 x 10^-139^	*Colletotrichum* sp. (MT196320.1)
BvFVII	OP604491	100%	100%	4 x 10^-137^	*Phomopsis* sp. (JN153066.1)
BvFVIII	OP604492	100%	100%	5 x 10^-146^	*Pestalotiopsis mangiferae* (MH244393.1)
BvFIX	OP604493	100%	98.53%	1 x 10^-131^	*Phomopsis* sp. (KC193052.1)

Oral antihyperglycemic thiazolidinediones favor insulin sensitization mediated by PPARγ activation. They have long been used for the treatment of type 2 diabetes, but several serious adverse effects, such as loss of bone density, retention of body fluids, troglitazone-related liver problems, and heart problems related to rosiglitazone consumption (Singh et al., 2007). However, PPARγ ligands remain an effective option in the treatment of type 2 diabetes. They can improve insulin sensitivity in all tissues by producing increased glucose uptake in peripheral tissues. Partial agonists may have more positive effects as PPARγ ligands in diabetes by decreasing adverse effects caused by the activation of unwanted transcription, favored by the binding of full agonists, and by preventing Cdk5-mediated PPARγ phosphorylation, improving insulin sensitivity. ERK can also phosphorylate serine 273 of PPARγ and can be suppressed by Cdk5 *via* MEK activation (Banks et al., 2015), and, in this way, PPARγ has its function controlled. Thus, the treatment of type 2 diabetes with ERK/MEK axis inhibitors could be used to improve insulin resistance.

To assess whether the extracts showed agonist activity on the different PPAR isoforms, we performed a gene reporter assay, followed by treatment with the different extracts at maximum concentrations established by the results of the cytotoxicity assay performed previously. Among the tested extracts, two of them, extracts BvFII and BvFVII, showed PPAR agonist activity (**Figures 2B, C**). Extract BvFII at a concentration of 20.16 µg.mL^-1^ (**Figure 2B**) activated all three PPAR isoforms (α, β/δ and γ), showing to be a pan-agonist. In turn, extract BvFVII activated only PPARα and PPARβ, but showed low activation on chimeric PPARγ (LBD-PPARγ/DBD-GAL4).

Then, it was evaluated whether extracts from fungi BvFII and BvFVII were able to modify the structure of the LBD of PPARγ. For this, we performed the protease protection assay. The LBDs of PPARs have a large binding pocket, the largest concerning other nuclear receptors. When binding to the agonist, LBD undergoes crucial changes in its structure, reflecting the difficulty of accessing proteolytic sites on its surface and, therefore, increasing its resistance to digestion by proteases. In this case, when analyzed by SDS-PAGE, it shows a typical running pattern on the polyacrylamide gel, with evidence of a band of approximately 25 kDa that, in the presence of an agonist, it resists degradation by proteases. The result presented shows that, when in the presence of extracts of fungi BvFII and BvFVII, the LBD of PPARγ presents electrophoretic patterns like each other, suggesting that these extracts protected the receptor more efficiently than rosiglitazone (**Figure 4A**). This is clear since the band of approximately 25 kDa appears more protected. Berger et al. (2003) describe a partial agonist that showed a pattern of protection against trypsin activity similar to that shown in these results. An assay was carried out to evaluate the proteolytic activity of trypsin in the presence of the extracts (**Figure 4B**) to rule out the hypothesis that some substance present in the extracts was preventing the proteolytic function of trypsin since there are studies that describe the production of proteases by fungi (De Souza et al., 2015). The results showed that there was no difference between the degradation pattern of gelatin in the presence or absence of extracts and vehicle (DMSO). This suggests that the effect of the extracts in the protection against protease digestion assay was by direct binding to PPARγ.

So far, some studies have shown the agonist activity of metabolites from fungi on PPARγ (Sato et al., 2002; Lee et al., 2008; Lee et al., 2012; Hsu et al., 2013) and another in inhibiting its expression, but, to our knowledge, none with activity on the other α and β/δ receptor isoforms. However, works showing the agonist activity of natural products from plants on PPARγ (Wang et al., 2014) and the other PPAR isoforms (α and β/δ) are also activated by natural products. Martin et al. (2013) showed four isoflavones (polyphenolic compounds) with agonist activity on PPARα, PPARβ/δ, and PPARγ, characterizing these substances as pan-agonists. In another work, it was shown that the hexane extract of *Tabebuia heptaphylla* has pan-agonist activity on PPARs. It has also been shown that PPARα can be activated by a compound isolated from tomatoes, 9-oxo-10(E),12(E)-octadecadienoic acid (Kim et al., 2011), and another isolate of *Cannabis sativa* (Nadal et al., 2017) Green tea extract, in turn, was able to activate PPARβ/δ (Danesi et al., 2009) while coffee extract inhibited PPARγ (Aoyagi et al., 2014).

TZDs can promote the differentiation of preadipocytes, causing unfavorable effects of weight gain in patients who use them (Wilding, 2006). Activation of PPARα also promotes preadipocyte differentiation. To assess whether this effect could also be caused by extracts from fungi BvFII and BvFVII, 3T3-L1 preadipocytes were induced to differentiate in the presence or absence of extracts, rosiglitazone, or vehicle (DMSO) (**Figure 3A**). The extracts from fungi BvFII and BvFVII stimulated a slight accumulation of lipids in the cells, visibly lower than that promoted by rosiglitazone at a concentration of 10^-5^ M. This result was unexpected since full PPARγ agonists are known to be capable of inducing adipogenesis in 3T3-L1 cells (Rosen and Spiegelman, 2014), and PPARα agonists influence 3T3-L1 preadipocyte.

Therefore, it was interesting to evaluate the influence of these extracts on gene transcription of the FABP4 (aP2) protein. The FABP4 (aP2) protein is a lipid chaperone that is located in the cell cytoplasm and is regulated by PPARγ in adipocytes, where it is highly expressed and involved in lipid storage. In the real-time PCR assay (**Figure 3D**) and the Western Blotting (**Figure 3C**), it was observed that they did not influence the gene and protein expression of FABP4 (aP2), confirming the result observed in the adipocyte differentiation assay and lipid disclosure by red oil O staining (**Figure 3A**).

One hypothesis to explain the low induction of adipogenesis by the extracts, even with positive agonist activity and suggestion of binding to the receptor, would be that the extracts were somehow hampering the action of aP2. The aP2 gene is flanked by the PPARγ response element, and the non-functional aP2 decreases the adipogenic effect. However, to confirm this hypothesis, other experiments are necessary.

These findings may contribute to the discovery of agonist molecules that can be tested for use as antihyperglycemic agents in the treatment of type 2 diabetes and dyslipidemia. With the molecular identification and phylogenetic analysis of the fungi, it was possible to verify that the extract of the fungi BvFII and BvFVII, but not the BvFIX of the genus *Phomopsis*, are the ones with the highest degree of kinship (**Figure 1**). This result may explain the way the extract of the fungus BvFIX didn’t show activity on PPARs assays.

Oxidative stress in the cell results from the imbalance between the antioxidant defense system, which is reduced, and the production of reactive oxygen species, which is increased. This imbalance is involved in the etiology of several diseases mediated, among other pathways, by damage to DNA, lipids, and proteins, in addition to signaling for deregulated gene transcription.

Flavonoids have also been described as agonists of different PPARs (Salam et al., 2008; Quang et al., 2013; Xu et al., 2018). However, flavonoids also have antioxidant and pro-oxidant activity, depending on their concentration in the reaction medium and the pH of the medium. The pro-oxidant effect of flavonoids is, as a rule, directly proportional to the total number of hydroxyl groups and is responsible for the cytotoxic and pro-apoptotic effects of flavonoids isolated from various medicinal plants (Metodiewa et al., 1999). These characteristics of flavonoids agree with the results obtained (**Figure 2**), in which extracts with the lowest polyphenol content were the ones that showed low antioxidant activity when tested with the stable radical DPPH. In a review written by Hamilton et al. (2012), endophytic fungi are important in the symbiotic relationship that exists between them and the host plants, and hyphal growth occurs more easily in leaves that are in growth. Initially, when interacting with the plant, it is necessary for the fungus to produce reactive oxygen species (ROS), thus preventing the plant’s defenses from acting. It is not known for sure what defines the relationship of symbiosis or parasitism, but after infection, there is a balance in the production of ROS and antioxidant substances by both the plant and the fungus, and this relationship is, in some cases, species-specific (Tanaka et al., 2006).

To further advance the understanding of the antioxidant mechanism of action of the extracts, they were evaluated for their ability to prevent lipid peroxidation and to scavenge the superoxide radical anion. The process of lipid peroxidation of polyunsaturated fatty acids depends on the presence of oxygen. These fatty acids can activate peroxisome proliferator-activated receptors α, β/δ, and γ. Antioxidant agents can act by preventing the oxidation of these fatty acids, and thus, the action of the pro-oxidant agent, or by inactivating radical substances. High concentrations of glucose increase the concentration of reactive oxygen species (ROS) and also the activation of an enzyme called phospholipase A2, which is capable of inducing the phosphorylation of two serines (505 and 515) (Pavicevic et al., 2008) and the release of arachidonic acid and linoleic acid from the cell membrane phospholipid. After release, these fatty acids are oxidized (lipid peroxidation) by ROS producing, in addition to other products, 4-hydroxyl-2-nonenal (4-HNE), which binds to PPARβ/δ, increasing insulin secretion. When chronic hyperglycemia occurs, 4-HNE is produced in excess, and adducts with proteins, DNA, and phospholipids are formed, with consequent β-cell apoptosis in the pancreas.

In the test performed, it was evaluated whether the extracts were able to prevent the beginning of the lipid peroxidation process. As the reaction occurs in an oil-in-water emulsion, it is necessary to consider the polarity of the antioxidant agent since nonpolar substances may present higher activity because they are retained in the oil droplets, where the fatty acid is located, in the case of the test, linoleic acid. Both BHT (2,6-bis(1,1-dimethylethyl)-4-methylphenol) and tocopherol are nonpolar substances and were used as positive controls in the assay. Except for the fungus BvFIII extract, all the extracts tested were able to prevent linoleic acid peroxidation (**Figure 2C**). The fact that extracts from fungi BvFII and BvFVII show agonist activity in PPARβ/δ and, in addition, are antioxidants, may represent an interesting activity when we think that this could prevent β-cell apoptosis and, thus, prevent the development of diabetes. The presence of low polar antioxidant substances, which were in emulsion interphase, or nonpolar miscible in the oil phase, may have been responsible for the good activity in the inhibition of lipid peroxidation. As shown in **Figure 2A**, the extracts have flavonoids. This class of substances has a lipophilic character and may be responsible for the antioxidant action. Regarding the fungus BvFIII extract, a hypothesis that explains its pro-oxidant activity is that the concentration of the active substance is very high, and this was the cause of the pro-oxidative activity, which resulted in the oxidation of linoleic acid. It is also known that flavonoids can have an antioxidant or pro-oxidant character, depending on the pH of the medium or the presence of a transition metal in the reaction medium, such as copper (Cu^2+^) or iron, in addition to oxygen (Cao et al., 1997).

To evaluate the capacity of scavenging the superoxide radical anion, ( ${\text{O}}_{2}^{-}\cdot$ ), by the extracts, the xanthine/xanthine oxidase system was used as a producer of ROS. It is known that the superoxide radical anion is present in aerobic cells, plays an essential role in oxidation reactions, and interferes with cell signaling, together with other reactive oxygen species. Furthermore, it is involved in the pathogenesis of several diseases. As in the lipid peroxidation assay, the presence of flavonoids in the extracts may explain the ability to scavenge the ${\text{O}}_{2}^{-}\cdot$ radical, highlighting the fungus BvFIII extract (**Figure 2D**), which was able to do so at a low concentration of 0.083 mg.mL^-1^. To understand the relevance of this result, in work published by Dong et al. (2013), the best activity in bee honey extract was observed at a concentration of 28 mg.mL^-1^. With this, it is possible to perceive that all extracts obtained showed excellent activity when compared to the cited work.

More studies are still needed for a better understanding of the metabolic pathways and integrated effects of the sequestration of reactive oxygen species and activation of nuclear receptors, but the results obtained can be considered promising since the same extracts showed the ability to activate PPARγ without much interference in adipocyte differentiation, and were still able to inhibit the initiation step of lipid peroxidation and scavenge the superoxide radical anion. These biological activities are promising in the search for candidates for new antihyperglycemic agents, as they would allow the development of drugs with specific activities, with fewer incidences of adverse effects.

## Data availability statement

The raw data supporting the conclusions of this article will be made available by the authors upon request.

## Author contributions

PM performed the experiments, analyzed the data, and wrote the manuscript. LA contributed with experiments of extracts obtention. FN coordinated the master project. MB conceived and coordinated this study. All authors contributed to the manuscript and approved the submitted version.
